# Comparison of ciprofol and propofol for endoscopic retrograde cholangio-pancreatography anesthesia: a systematic review and meta-analysis

**DOI:** 10.3389/fphar.2025.1592781

**Published:** 2025-08-15

**Authors:** Kai Wu, Min Liao, Juan Deng, Yunfeng Yu, Yuman Yin, Xinyu Yang, Rong Yu, Zhenjie Liu

**Affiliations:** ^1^ Department of Gastroenterology, The First Hospital of Hunan University of Chinese Medicine, Changsha, Hunan, China; ^2^ Department of Anesthesiology, People’s Hospital of Ningxiang City, Changsha, Hunan, China; ^3^ School of Traditional Chinese Medicine, Hunan University of Chinese Medicine, Changsha, Hunan, China

**Keywords:** ciprofol, endoscopic retrograde cholangio-pancreatography, gastrointestinal endoscopy, perioperative complications, meta-analysis, trial sequential analysis

## Abstract

**Objective:**

The potential of ciprofol in endoscopic anesthesia is receiving increasing attention. Compared to propofol, ciprofol exhibits stronger sedative effects and requires a lower dosage. This study aimed to compare the safety of ciprofol and propofol in Chinese patients undergoing endoscopic retrograde cholangio-pancreatography (ERCP) anesthesia.

**Methods:**

A comprehensive literature search was conducted across eight common databases before 1 January 2025, including PubMed, Embase, the Cochrane Library, and Web of Science, China National Knowledge Infrastructure, China Science and Technology Journal Database, WanFang, and SinoMed. After screening the literature according to established standards, the meta-analysis and trial sequential analysis (TSA) were conducted using Review Manager 5.3 and TSA 0.9.5.10 beta, respectively. Finally, publication bias for each outcome was assessed using Harbord regression analysis.

**Results:**

Seven randomized controlled trials (RCTs) with 1,264 participants undergoing ERCP were included, and all included studies were conducted in China, with participants representing the Chinese population. The meta-analysis showed that compared to propofol, ciprofol reduced bradycardia (risk ratio [RR] 0.44, 95% confidence interval [CI] 0.26–0.76, P = 0.003, n = 4), hypotension (RR 0.72, 95% CI 0.55–0.95, P = 0.02, n = 4), respiratory depression (RR 0.25, 95% CI 0.14–0.44, P < 0.00001, n = 5), hypoxemia (RR 0.35, 95% CI 0.21–0.58, P < 0.0001, n = 5), and injection pain (RR 0.17, 95% CI 0.11–0.26, P < 0.00001, n = 7), but had no significant effect on choking cough, involuntary movements, or nausea and vomiting. TSA showed a conclusive benefit for bradycardia, respiratory depression, hypoxemia, and injection pain, whereas the benefit for hypotension needs further validation. Harbord regression analysis showed no publication bias for any of the outcomes, except for hypotension.

**Conclusion:**

Compared with propofol, ciprofol has been shown to reduce the incidence of bradycardia, respiratory depression, hypoxemia, and injection pain in patients undergoing ERCP; however, its effect on the occurrence of hypotension still requires further investigation. Future studies are warranted to clarify the safety, efficacy, and optimal dosing of ciprofol across various patient populations, particularly those with complex comorbidities. These efforts would facilitate the broader application of ciprofol in ERCP and other surgical procedures, such as gastrointestinal and ophthalmic surgeries.

**Systematic review registration:**

www.crd.york.ac.uk/PROSPERO/view/CRD420251090047, identifer, CRD420251090047

## 1 Introduction

Endoscopic retrograde cholangio-pancreatography (ERCP) is a highly efficient and minimally invasive diagnostic and treatment method widely used to diagnose and treat pancreatic and biliary diseases such as acute cholangitis, choledocholithiasis, acute and chronic pancreatitis, pancreatic calculi, and pseudocysts (Khamaysi and Taha, 2020; Schmalz and Geenen, 1999). However, ERCP is highly invasive and often associated with several adverse events, including pain, nausea, vomiting, bleeding, and perforation (Talukdar, 2016; Pal and Ramchandani, 2024). To mitigate the negative impact of surgery on patients, the Consensus Guidelines for the Perioperative Management of Patients Undergoing ERCP recommend deep sedation (Azimaraghi et al., 2023). Among the various sedatives and anesthetics, propofol is widely used in ERCP due to its rapid onset and metabolism (Cheriyan and Byrne, 2014). However, the risk of respiratory and circulatory depression associated with propofol anesthesia continues to raise concerns among anesthesiologists (Cheriyan and Byrne, 2014). These risks are particularly pronounced in the older population, with a clear dose-dependent relationship (Short and Bufalari, 1999; Shimizu et al., 2021; Phillips et al., 2015). Worryingly, most patients undergoing ERCP are older, and they may experience severe propofol-induced respiratory and circulatory depression during ERCP (Rastogi et al., 2006). Therefore, anesthesiologists are eager to find an alternative to propofol that minimizes the risk of respiratory and circulatory depression, while achieving rapid and effective sedation.

Ciprofol is a novel short-acting intravenous anesthetic with a structure and function similar to those of propofol (Task F orce on Gui et al., 2023). Although both act by binding to the gamma-aminobutyric acid (GABA) receptors, ciprofol is four to five times more effective than propofol and causes less respiratory and circulatory depression (Lu et al., 2023). Due to its higher affinity for the GABA receptors, the effective dosage needed for ciprofol is significantly lower than that of propofol. Additionally, the lower aqueous-phase drug concentration of ciprofol does not cause the same severe injection pain as propofol (Qin et al., 2017). Because of these pharmacological properties, ciprofol has a stronger anesthetic effect and better safety profile than propofol. However, previous studies have yielded controversial results regarding whether ciprofol is a safer anesthetic agent than propofol during ERCP. Some studies found that ciprofol significantly reduced respiratory and circulatory adverse events compared to propofol (Wang et al., 2024), while other studies reported no beneficial effects of ciprofol on outcomes such as hypoxemia and hypotension (Ding et al., 2024). Moreover, owing to the lack of relevant systematic reviews and meta-analyses, it is unclear whether ciprofol has the potential to replace propofol during ERCP. A rigorous synthesis of the existing randomized controlled trials (RCTs) is urgently needed to resolve these controversies and provide high-level evidence for clinical decision-making. Therefore, this study aimed to determine the comparative safety of ciprofol versus propofol during ERCP anesthesia through a meta-analysis and trial sequential analysis (TSA).

## 2 Methods

This study strictly followed the Preferred Reporting Items for Systematic Reviews and Meta-Analyses (PRISMA) guidelines and was registered in Prospective Register of Systematic Reviews (PROSPERO) (CRD420251090047) (Page et al., 2021).

### 2.1 Literature search

The relevant studies were retrieved from PubMed, Embase, the Cochrane Library, and Web of Science, as well as Chinese databases such as the China National Knowledge Infrastructure (CNKI), China Science and Technology Journal Database (CSTJ), WanFang, and SinoMed. The search fields were set to Title/Abstract, with the search strategy defined as follows: (Ciprofol OR Cyclopropanes OR HSK3486) AND (Endoscopic Retrograde cholangio-pancreatography OR ERCP OR Endoscopic Retrograde Cholangiopancreatographies OR Endoscopy OR Endoscope OR Endoscopies OR Endoscopic OR Gastroscope OR Gastroscopy OR Colonoscope OR Colonoscopy OR Sigmoidoscopy). The search was conducted from the inception of the database until 1 January 2025, without language or other restrictions.

### 2.2 Inclusion and exclusion criteria

Inclusion criteria: (i) RCTs; (ii) Participants underwent ERCP; (iii) Experimental group received ciprofol anesthesia and the control group received propofol anesthesia; and (iv) Cardiovascular outcomes, including bradycardia and hypotension; respiratory outcomes, including respiratory depression, hypoxemia, and choking cough; neurological outcomes, including injection pain and involuntary movements; and gastrointestinal outcomes, including nausea and vomiting.

Exclusion criteria: (i) Studies with duplicate publications; (ii) Reviews, animal experiments, and editorials; (iii) Studies lacking baseline data; and (iv) Studies with unavailable data.

### 2.3 Literature screening

The collected articles were imported into Zotero 7.0. Essential details such as titles, authors, journal names, volumes, issue numbers, and DOIs were manually verified to eliminate any remaining duplicates. The titles and abstracts of each article were reviewed by applying predefined inclusion and exclusion criteria. Subsequently, full texts of the remaining articles were reviewed. Each stage of the literature screening process was independently conducted by JD and YY, and their findings were cross-checked.

### 2.4 Data collection

Excel 2010 was used to create tables to record the basic characteristics and outcome data of each included study. The basic characteristic table included the first author, publication year, participant source, sample size, male ratio, average age, body mass index (BMI), American Society of Anesthesiologists (ASA) classification, anesthesia induction, and type of examination. Data related to the outcomes were recorded in the data statistical table. Each step was independently completed and cross-checked by JD and YY.

### 2.5 Risk assessment of bias

The Cochrane Bias Risk Assessment Tool was used to assess the methodological quality of the included articles. This tool includes items such as random sequence generation, allocation concealment, blinding of participants and personnel, blinding of outcome assessment, incomplete outcome data, selective reporting, and other biases, each categorized as low, unclear, or high risk. JD and YY independently completed and cross-checked the risk of bias assessment.

### 2.6 Statistical analysis

Review Manager software (version 5.3) was used to conduct the meta-analyses, sensitivity analyses, and publication bias assessments. First, the risk ratio (RR) was considered to indicate the effect size for dichotomous variables, while the I^2^ statistic was used to assess heterogeneity. An I^2^ value of <50% indicated no significant heterogeneity, leading to the use of a fixed-effect model for the meta-analysis; when I^2^ was ≥50%, indicating high heterogeneity, a random-effect model was applied. Statistical significance for the meta-analysis was set at P < 0.05. Second, leave-one-out sensitivity analysis was performed to explore the sources of heterogeneity and assess the robustness of the results. If the effect size and significance remained stable, the meta-analysis results were considered robust. Third, subgroup analyses were conducted based on the male ratio, average age, ASA classification, BMI, and surgical duration to assess the impact of these clinical factors on heterogeneity. Additionally, a meta-regression analysis was performed to further investigate the sources of heterogeneity when the number of studies included for a particular outcome exceeded ten. The covariates examined included age, BMI, ASA classification, and ciprofol dosage. Fourth, Harbord regression analysis was used to evaluate publication bias. When P ≥ 0.05 in Harbord regression analysis, it indicated no potential publication bias. Fifth, the TSA 0.9.5.10 Beta software was used to perform the TSA. A type I error rate of 5% and type II error rate of 20% were set, and the relative risk reduction was calculated based on the included studies. If the cumulative Z-curve crossed the trial sequential monitoring boundary, the existing data were considered adequate to draw conclusive decisions. Finally, the “Grading of Recommendations, Assessment, Development, and Evaluation” (GRADE) guideline was used to assess the certainty of evidence. The evaluation modules included the risk of bias, inconsistency, indirectness, imprecision, and publication bias.

## 3 Results

### 3.1 Literature screening process

**FIGURE 1:**
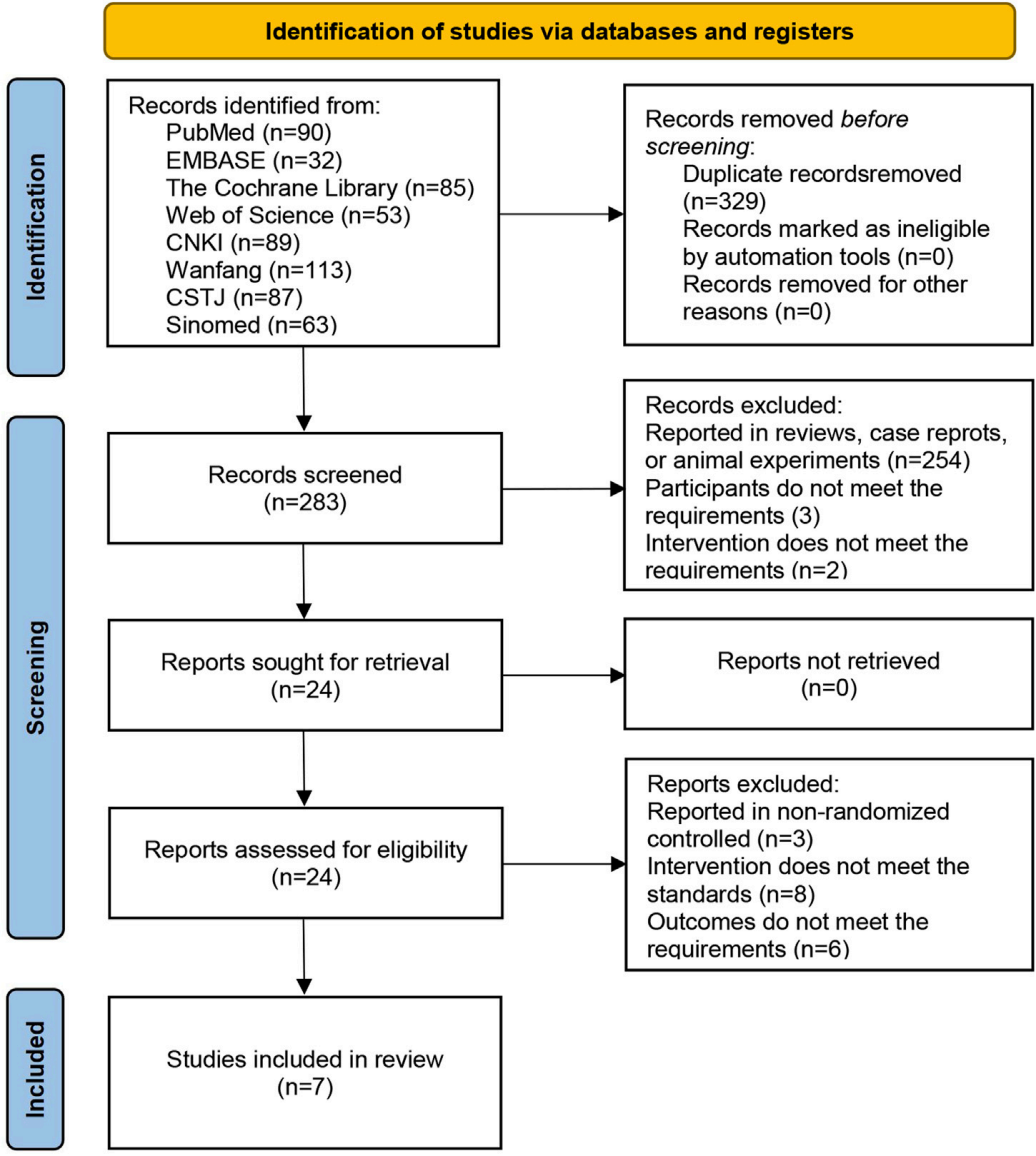
Flowchart of literature screening.

A total of 612 articles were retrieved from eight databases. During the screening process, 329 articles were excluded because of duplication, and 259 articles were excluded as they were irrelevant to the topic. Subsequently, 17 articles were excluded after full-text review because they failed to meet the inclusion criteria. Among these, three articles reported non-RCTs, eight articles reported inconsistent interventions, and six articles provided unusable indicators. Ultimately, seven articles were included in the analysis, as shown in Figure 1.

### 3.2 Basic characteristics of the included studies

The meta-analysis included seven RCTs (Wang et al., 2024; Ding et al., 2024; Mo, 2024; Zhao et al., 2023; Gao and Liu, 2024; He et al., 2024; Shen et al., 2024) involving 1,264 patients undergoing ERCP. Among them, 657 patients received ciprofol anesthesia and 607 received propofol anesthesia. The studies were published between 2020 and 2024, and all were conducted in China. The participants were all Chinese, with an average male proportion of 52.8%, average age of 65.8 years, average BMI of 22.7 kg/m^2^, and average ASA III ratio of 39.7%. The induction dose of ciprofol ranged from 0.3 mg/kg to 0.5 mg/kg, with the maintenance dose ranging from 0.6 mg/(kg·h) to 1.5 mg/(kg·h). For propofol, the induction dose ranged from 0.5 mg/kg to 2.0 mg/kg, and the maintenance dose ranged from 1.5 mg/(kg·h) to 12 mg/(kg·h) (Table 1).

**TABLE 1:** Basic characteristics of the included studies.

Author name	Patient number	Sample	Male /%	Age /years	BMI /(kg·m^-2^)	ASA III /%	Surgical duration /minutes	Anesthesia Induction	Anesthesia Maintenance
Ding et al. (2024)	284	142	51%	73.6	23.1	44%	47.1	C 0.3–0.4 mg/kg	C 0.8–1.2 mg/(kg·h)
		142	51%	73.7	22.3	44%	48.2	P 1.5–2.0 mg/kg	P 4–12 mg/(kg·h)
Gao and Liu. (2024)	100	50	56%	73.4	23.6	/	/	C 0.3 mg/kg	C 1.0–1.5 mg/(kg·h)
		50	58%	73.3	23.6	/	/	P 1.5 mg/kg	P 4.0–12.0 mg/(kg·h)
He et al. (2024)	150	50	44%	50.0	22.6	0%	44.8	C 0.4 mg/kg	C 1.0 mg/(kg·h)
		50	48%	52.1	23.0	0%	45.3	C 0.5 mg/kg	C 1.5 mg/(kg·h)
		50	50%	51.4	23.5	0%	45.2	P 2.0 mg/kg	P 6.0 mg/(kg·h)
Mo. (2024)	60	30	57%	70.7	21.7	30%	28.2	C 0.2 mg/kg	/
		30	53%	69.9	21.3	27%	27.3	P 1.0 mg/kg	/
Shen et al. (2024)	80	40	65%	>65.0	22.3	0%	/	C 0.3–0.5 mg/kg	C 1.0–1.5 mg/(kg·h)
		40	68%	>65.0	22.7	0%	/	P 1.5–2.0 mg/kg	P 2.0–5.0 mg/(kg·h)
Wang et al. (2024)	306	153	50%	55.0	22.9	33%	/	C 0.2 mg/kg	C 0.6–0.8 mg/(kg·h)
		153	56%	54.8	22.4	40%	/	P 1.0 mg/kg	P 4.0–6.0 mg/(kg·h)
Zhao et al. (2023)	284	142	51%	73.6	23.1	75%	27.8	C 0.3–0.4 mg/kg	C 1.0–1.5 mg/(kg·h)
		142	51%	73.6	22.3	73%	27.6	P 1.5–2.0 mg/kg	P 4.0–12.0 mg/(kg·h)

There were no significant differences in the male to female ratio, age, BMI, or ASA III, ratio between the experimental and control groups in each included study. C, ciprofol; P, propofol, BMI, body mass index; ASA, american society of anesthesiologists.

### 3.3 Risk assessment of bias

**FIGURE 2:**
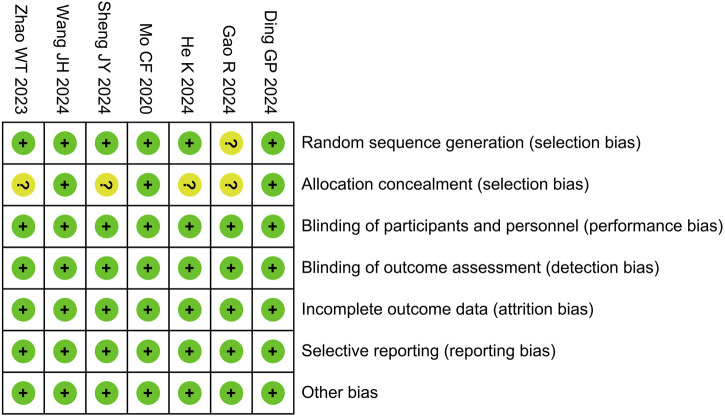
Risk of bias summary.

In the Cochrane risk of bias assessment, one study was rated as having an unclear risk for random sequence generation due to the absence of a description of the randomization method in its manuscript. Without this information, it is impossible to accurately determine whether the random sequence generation was conducted properly, resulting in an unclear risk rating. Regarding allocation concealment, four studies were also rated as having an unclear risk because they provided no description of the concealment methods used. When the concealment method is not specified, we cannot be certain that participants and researchers were unaware of the upcoming allocations, which complicates the assessment of the true risk of bias in this domain. However, all other domains were assessed as low risk, as illustrated in Figure 2.

### 3.4 Meta analyses

#### 3.4.1 Circulatory system outcomes

**FIGURE 3:**
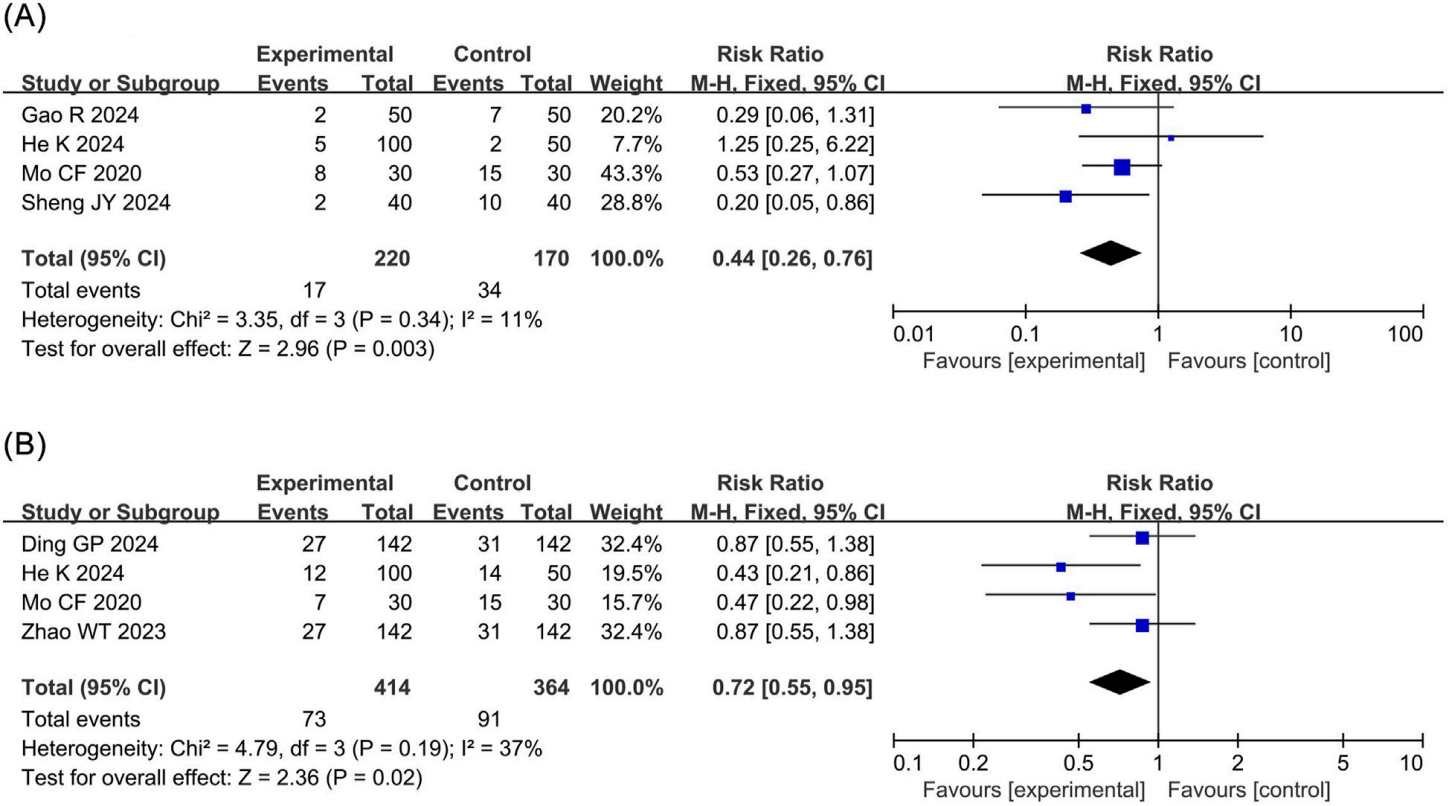
Forest plots of meta-analyses of cardiovascular outcomes. **(A)** Bradycardia **(B)** Hpotension.

Bradycardia: The meta-analysis of bradycardia included four RCTs (Mo, 2024; Gao and Liu, 2024; He et al., 2024; Shen et al., 2024) involving 390 patients. The results revealed that compared to propofol, ciprofol significantly reduced the risk of bradycardia by 56% in patients undergoing ERCP (RR 0.44, 95% confidence interval [CI] 0.26–0.76, P = 0.003, I^2^ = 11%), as illustrated in Figure 3A.

Hypotension: The meta-analysis of hypotension encompassed four RCTs (Ding et al., 2024; Mo, 2024; Zhao et al., 2023; He et al., 2024) comprising 778 patients. The results demonstrated that compared to propofol, ciprofol significantly reduced the incidence of hypotension by 28% in patients undergoing ERCP (RR 0.72, 95% CI 0.55–0.95, P = 0.02, I^2^ = 37%), as shown in Figure 3B.

#### 3.4.2 Respiratory system outcomes

**FIGURE 4:**
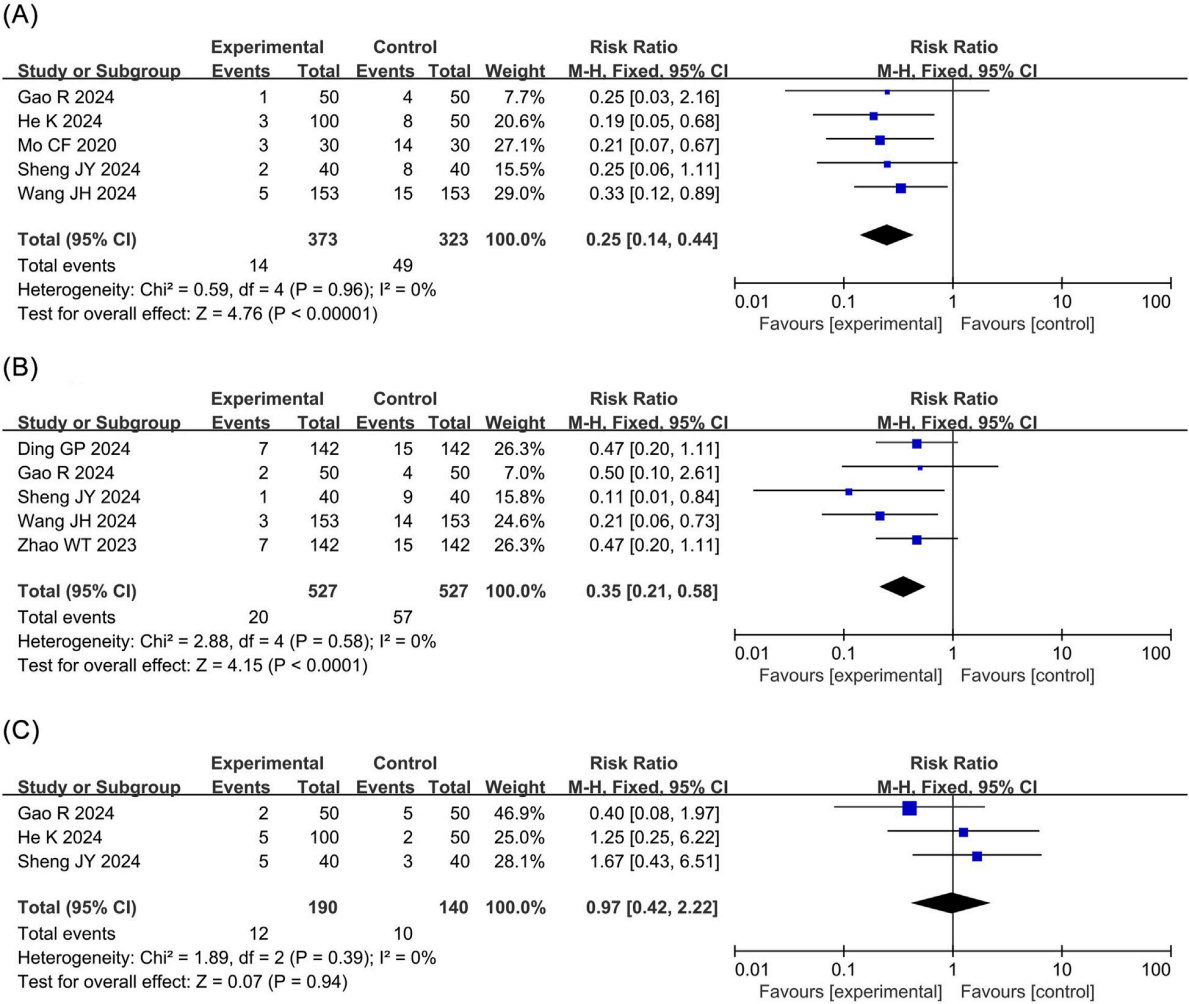
Forest plots of meta-analyses of respiratory outcomes. **(A)** Respiratory depression **(B)** Hypoxemia **(C)** Choking cough.

Respiratory depression: The meta-analysis of respiratory depression included five RCTs (Wang et al., 2024; Mo, 2024; Gao and Liu, 2024; He et al., 2024; Shen et al., 2024) comprising 696 patients. The findings indicated that compared to propofol, ciprofol substantially reduced the incidence of respiratory depression by 75% in patients undergoing ERCP (RR 0.25, 95% CI 0.14–0.44, P < 0.00001, I^2^ = 0%), as depicted in Figure 4A.

Hyoxemia: The meta-analysis of hypoxemia included five RCTs (Ding et al., 2024; Wang et al., 2024; Zhao et al., 2023; Gao and Liu, 2024; Shen et al., 2024) with 1,054 patients. The results indicated that compared to propofol ciprofol significantly decreased the incidence of hypoxemia by 65% in patients undergoing ERCP (RR 0.35, 95% CI 0.21–0.58, P < 0.0001, I^2^ = 0%), as presented in Figure 4B.

Choking cough: The meta-analysis of choking cough included three RCTs (Gao and Liu, 2024; He et al., 2024; Shen et al., 2024) involving 330 patients. The results revealed that there was no statistical difference in the incidence of choking cough between the ciprofol and propofol groups (RR 0.97, 95% CI 0.42–2.22, P = 0.94, I^2^ = 0%), as shown in Figure 4C.

#### 3.4.3 Nervous and digestive system outcomes

**FIGURE 5:**
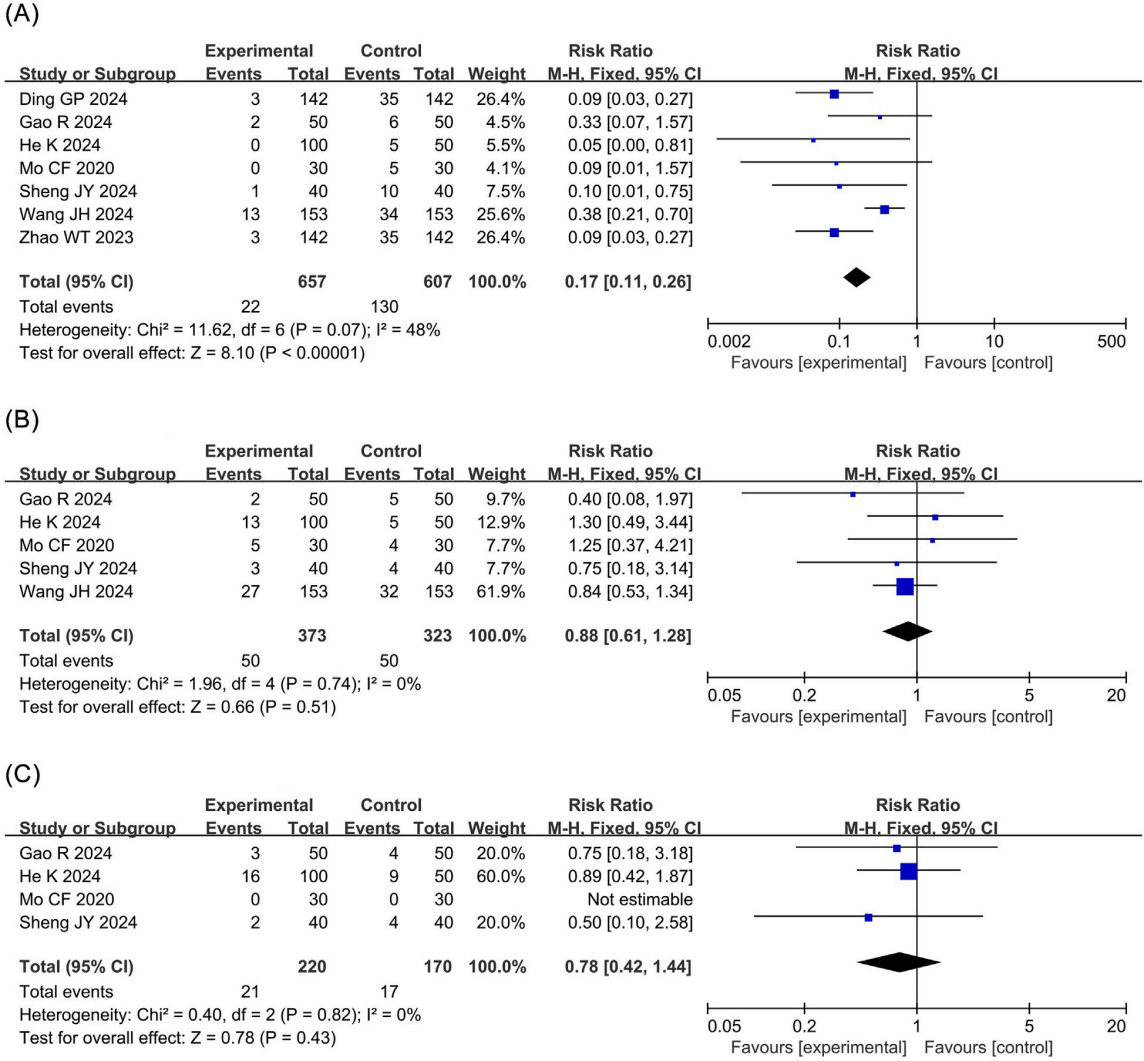
Forest plots of meta-analyses of neurological and gastrointestinal outcomes. **(A)** Injection pain **(B)** Involuntary movement **(C)** Nausea and vomiting.

Injection pain: The meta-analysis of injection pain incorporated seven RCTs (Wang et al., 2024; Ding et al., 2024; Mo, 2024; Zhao et al., 2023; Gao and Liu, 2024; He et al., 2024; Shen et al., 2024) with 1,264 patients. The results revealed that compared to propofol, ciprofol significantly reduced the incidence of injection pain by 83% in patients undergoing ERCP (RR 0.17, 95% CI 0.11–0.26, P < 0.00001, I^2^ = 48%), as shown in Figure 5A.

Involuntary movement: The meta-analysis of involuntary movements included five RCTs (Wang et al., 2024; Mo, 2024; Gao and Liu, 2024; He et al., 2024; Shen et al., 2024) comprising 696 patients. The results demonstrated no statistical difference in the incidence of involuntary movements between the ciprofol and the propofol groups (RR 0.88, 95% CI 0.61–1.28, P = 0.51, I^2^ = 0%), as depicted in Figure 5B.

Nausea and vomiting: The meta-analysis of nausea and vomiting included four RCTs (Mo, 2024; Gao and Liu, 2024; He et al., 2024; Shen et al., 2024) involving 390 patients. No statistical difference was detected in the incidence of nausea and vomiting between the ciprofol and the propofol groups (RR 0.78, 95% CI 0.42–1.44, P = 0.43, I^2^ = 0%), as presented in Figure 5C.

### 3.5 Sensitivity analysis

In the aforementioned meta-analysis, moderate heterogeneity was observed in the results concerning hypotension and injection pain. Therefore, the sources of this heterogeneity were explored using leave-one-out sensitivity analysis. The results revealed that the heterogeneity in hypotension originated from the study by He et al. (2024) and was attributed to differences in the average age. After excluding the study by He et al., the difference between the ciprofol and propofol groups for hypotension was no longer significant (RR 0.79, 95% CI 0.59–1.07, P = 0.12, I^2^ = 13%). Additionally, the heterogeneity in injection pain was attributed to the study by Wang et al. (2024) and was due to variations in the dose of ciprofol. Specifically, Wang et al. (2024) employed a maintenance dose of 0.6–0.8 mg/(kg·h), which was significantly lower than the doses used in other studies (Ding et al., 2024; Mo, 2024; Zhao et al., 2023; Gao and Liu, 2024; He et al., 2024; Shen et al., 2024). After excluding Wang et al.’s study (Wang et al., 2024), the difference in injection pain between the ciprofol and propofol groups remained statistically significant (RR 0.10, 95% CI 0.05–0.19, P < 0.00001, I^2^ = 0%). However, due to the absence of clear stratification based on the induction and maintenance doses of ciprofol, further subgroup analyses to evaluate the effects of different dosage regimens were not feasible.

Moreover, the robustness of each outcome was evaluated using leave-one-out sensitivity analysis, which demonstrated that the results for bradycardia, respiratory depression, hypoxemia, choking, cough, injection pain, involuntary movements, and nausea and vomiting were all robust. However, the result for hypotension was not robust, as its statistical significance disappeared after excluding the study by He et al. (2024) (RR 0.79, 95% CI 0.59–1.07, P = 0.12, I^2^ = 13%). Unfortunately, despite the initial plan to conduct a meta-regression analysis to further explore the sources of heterogeneity, it could not be performed due to the inclusion of fewer than ten studies.

### 3.6 Subgroup analysis

Subgroup analysis was conducted to evaluate the impact of clinical factors such as male ratio, average age, ASA classification, BMI, and surgical duration on the heterogeneity and significance of hypotension and injection pain. Due to the lack of clear stratification for male ratio and BMI, corresponding subgroup analyses were not performed. Ultimately, we investigated the effects of average age, ASA classification, and surgical duration on clinical heterogeneity, as shown in Table 2.

**TABLE 2:** Subgroup analyses of hypotension and injection pain.

Outcome	Subject	Subgroup	I^2^/%	RR (95% CI)	P Value
Hypotension	Average age	≥60 ears	13	0.79 (0.59, 1.07)	0.12
		<60 years	0	0.43 (0.21, 0.86)	0.02
Injection pain	Average age	≥60 years	0	0.10 (0.05, 0.20)	<0.00001
		<60 years	52	0.32 (0.18, 0.57)	<0.0001
Hypotension	ASA I ratio	≥30%	0	0.87 (0.63, 1.21)	0.41
		<30%	0	0.45 (0.27, 0.74)	0.002
Injection pain	ASA I ratio	≥30%	78	0.15 (0.05, 0.50)	0.002
		<30%	0	0.08 (0.02, 0.33)	0.0005
Hypotension	Surgical duration	≥30 min	64	0.64 (0.32, 1.28)	0.21
		<30 min	49	0.74 (0.50, 1.09)	0.13
Injection pain	Surgical duration	≥30 min	0	0.08 (0.03, 0.23)	<0.00001
		<30 min	0	0.09 (0.03, 0.25)	<0.00001

Regarding average age, compared to propofol, ciprofol significantly reduced the incidence of hypotension in the subgroup of patients aged <60 years (RR 0.43, 95% CI 0.21–0.86, P = 0.02, I^2^ = 0%), while there was no significant effect on hypotension in the subgroup aged ≥60 years (RR 0.79, 95% CI 0.59–1.07, P = 0.12, I^2^ = 13%). In terms of ASA I ratio, ciprofol significantly decreased hypotension in the subgroup with ASA I ratio <30% (RR 0.45, 95% CI 0.27–0.74, P = 0.002, I^2^ = 0%), but had no significant effect on hypotension in the ASA I ratio ≥30% subgroup (RR 0.87, 95% CI 0.63–1.21, P = 0.41, I^2^ = 0%). For surgical duration, ciprofol did not significantly affect hypotension in either the subgroup with surgical duration <30 min (RR 0.74, 95% CI 0.50–1.09, P = 0.13, I^2^ = 49%) or the subgroup with surgical duration ≥30 min (RR 0.64, 95% CI 0.32–1.28, P = 0.21, I^2^ = 64%). These analyses suggest that the heterogeneity of hypotension may be related to average age and ASA I ratio, and the meta-analysis result of hypotension is not robust.

In terms of injection pain, compared to propofol, ciprofol significantly reduced the incidence of injection pain in both the subgroup of patients aged <60 years (RR 0.32, 95% CI 0.18–0.57, P < 0.0001, I^2^ = 52%) and the subgroup aged ≥60 years (RR 0.10, 95% CI 0.05–0.20, P < 0.00001, I^2^ = 0%). Regarding ASA I ratio, ciprofol significantly decreased injection pain in the subgroup with ASA I ratio < 30% (RR 0.08, 95% CI 0.02–0.33, P = 0.0005, I^2^ = 0%) and in the subgroup with ASA I ratio ≥30% (RR 0.15, 95% CI 0.05–0.50, P = 0.002, I^2^ = 78%). For surgical duration, ciprofol significantly reduced injection pain in both the subgroup with surgical duration < 30 min (RR 0.09, 95% CI 0.03–0.25, P < 0.00001, I^2^ = 0%) and the subgroup with surgical duration ≥30 min (RR 0.08, 95% CI 0.03–0.23, P < 0.00001, I^2^ = 0%). These analyses indicate that the heterogeneity of injection pain may be associated with surgical duration and is robust.

### 3.7 TSA

**FIGURE 6:**
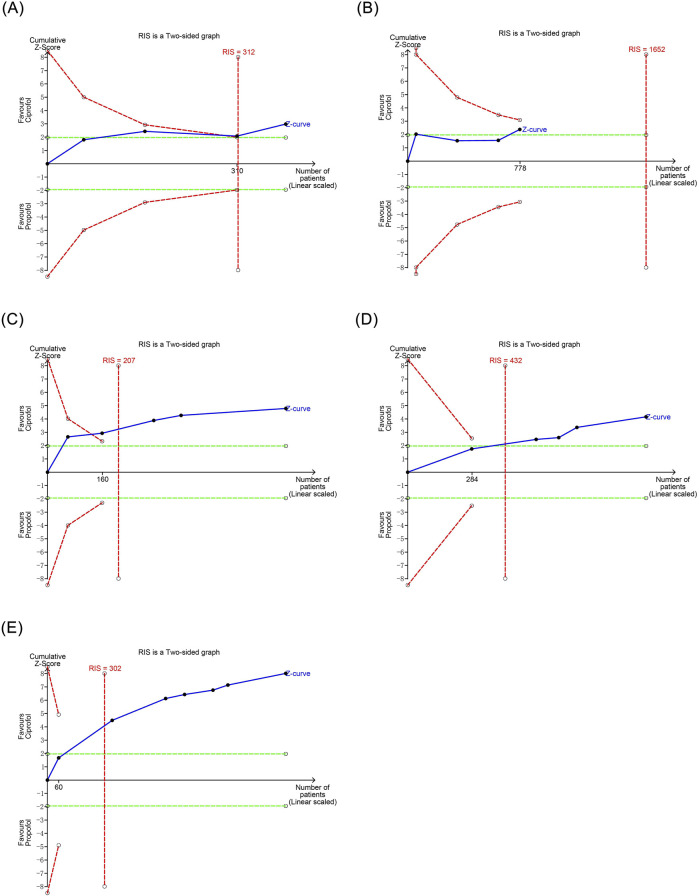
Trial sequential analysis results of positive outcomes. **(A)** Bradycardia **(B)** Hpotension **(C)** Respiratory depression **(D)** Hypoxemia **(E)** Injection pain.

TSA was conducted for secondary evaluation of the meta-analysis results that exhibited statistical significance. The TSA demonstrated that the cumulative Z-curve for bradycardia reached the trial sequential monitoring boundary in the third study, and the cumulative Z-curves for respiratory depression, hypoxemia, and injection pain reached the trial sequential monitoring boundary in the second study, indicating that these outcomes were conclusive. However, the TSA revealed that the cumulative Z-curve for hypotension had not yet reached the trial sequential monitoring boundary, suggesting that the results observed with the current sample size were not definitive and require further studies to assess the differences in the risk of hypotension with ciprofol and propofol, as shown in Figure 6.

### 3.8 Publication bias

**FIGURE 7:**
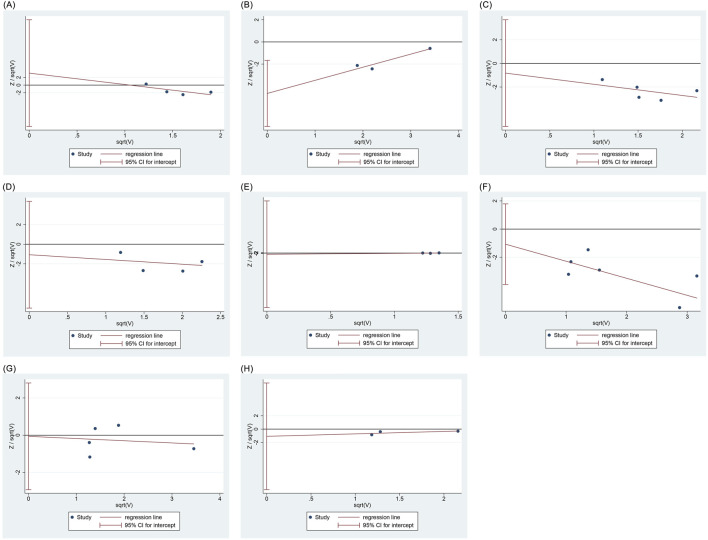
Regression analysis of publication bias. **(A)** Bradycardia **(B)** Hpotension **(C)** Respiratory depression **(D)** Hypoxemia **(E)** Choking cough **(F)** Injection pain **(G)** Involuntary movement **(H)** Nausea and vomiting.

Harbord regression analysis indicated no publication bias for bradycardia (P = 0.435), respiratory depression (P = 0.603), hypoxemia (P = 0.576), choking cough (P = 0.826), injection pain (P = 0.380), involuntary movements (P = 0.951), or nausea and vomiting (P = 0.334); however, hypotension demonstrated potential publication bias (P = 0.022), as shown in Figure 7.

### 3.9 Certainty of evidence

The GRADE guideline showed moderate certainty of evidence for bradycardia, respiratory depression, hypoxemia, and injection pain; low certainty of evidence for choking cough, involuntary movements, and nausea, and vomiting; and very low certainty of evidence for hypotension (Table 3).

**TABLE 3:** Certainty of evidence.

Outcome	Risk of bias	Inconsistency	Indirectness	Imprecision	Others	Certainty of evidence
Bradycardia	Serious	None	None	None	None	Moderate
Hypotension	Serious	None	None	Serious	Publication bias	Very Low
Respiratory depression	Serious	None	None	None	None	Moderate
Hypoxemia	Serious	None	None	None	None	Moderate
Choking cough	Serious	None	None	Serious	None	Low
Injection pain	Serious	None	None	None	None	Moderate
Involuntary movement	Serious	None	None	Serious	None	Low
Nausea and vomiting	Serious	None	None	Serious	None	Low

## 4 Discussion

### 4.1 Research background and findings

Although propofol is widely used for anesthesia induction and maintenance during ERCP, the respiratory and circulatory risks it poses remain a concern for anesthesiologists. Since its introduction, ciprofol, a derivative of propofol, has garnered significant attention from the anesthesiology community. Both ciprofol and propofol exert their sedative effects by binding to GABA receptors, with ciprofol exhibiting an affinity for these receptors that is 4–5 times greater than that of propofol (Lu et al., 2023). From a pharmacokinetic perspective, ciprofol has a higher lipophilicity, allowing it to distribute extensively into adipose tissue after entering the bloodstream (Qin et al., 2017). Research indicates that the concentration of ciprofol in brain tissue is 3.2 times higher than that in plasma, suggesting that ciprofol can cross the blood-brain barrier and exert central inhibitory effects (Liao et al., 2022). This results in a significantly lower required dose for intravenous administration of ciprofol compared to propofol at equivalent levels of sedation. Due to differences in receptor affinity and lipophilicity, the safety of these two drugs should be assessed based on their pharmacological characteristics and clinical effects rather than through direct dosage comparisons.

**FIGURE 8:**
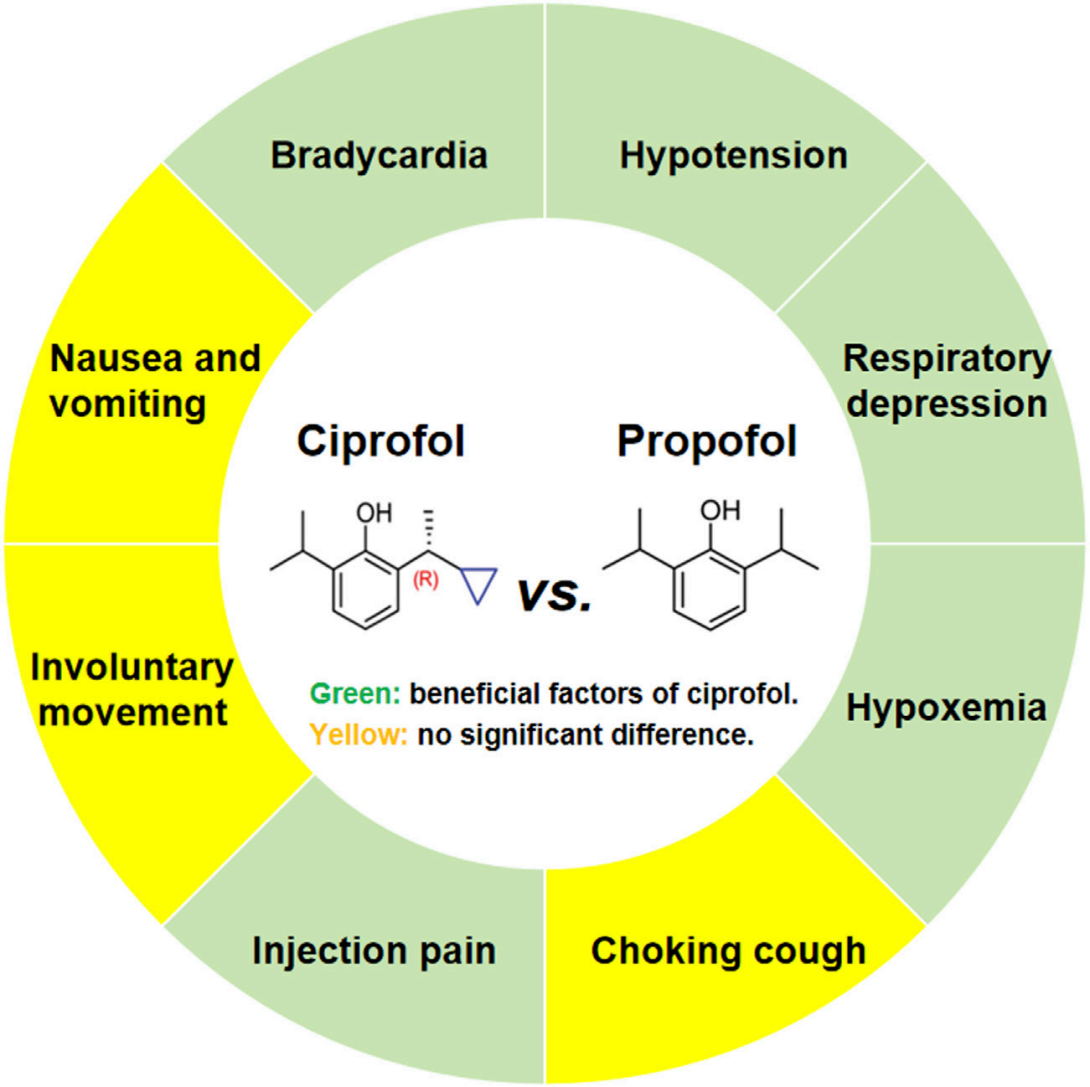
Main findings of the systematic review and meta-analysis.

While several previous meta-analyses have reported on the safety of ciprofol compared to propofol, they have primarily focused on endoscopic sedation and general anesthesia, without specifically addressing ERCP (Chen et al., 2025; Cheng et al., 2025; Liu et al., 2024; Qi et al., 2024). In fact, ERCP is a complex endoscopic procedure that differs significantly from standard gastrointestinal endoscopy, characterized by greater stimulation and longer procedure times. Therefore, this study focuses on the safety of ciprofol in the context of ERCP, aiming to accurately assess the value of ciprofol as a replacement for propofol in this setting. The findings suggest that compared to propofol, ciprofol significantly reduces the incidence of bradycardia, hypotension, respiratory depression, hypoxemia, and injection pain, with no significant effect on the incidence of choking cough, involuntary movements, or nausea and vomiting (Figure 8).

### 4.2 Effects on the circulatory system

The meta-analysis revealed that compared to propofol, ciprofol significantly reduced the incidence of bradycardia and hypotension. Subsequent TSA indicated that the benefit of ciprofol in reducing bradycardia was conclusive; In prior meta-analyses, Chen et al. (2025) and Cheng et al. (2025) reported that ciprofol reduced the incidence of bradycardia in older patients during the perioperative period (RR 0.64, 95% CI 0.48–0.85) and gastrointestinal endoscopy (odds ratio [OR] 0.66, 95% CI 0.49–0.87), supporting our findings. The differences in the incidence of bradycardia between ciprofol and propofol may be related to their pharmacological effects. Firstly, propofol has a slower metabolic rate, particularly in older patients, which can lead to drug accumulation in the body and prolong its effects, thereby increasing the risk of bradycardia (Huang et al., 2023). In contrast, ciprofol is metabolized more rapidly, resulting in less accumulation in the body, allowing for quicker clearance and reducing the risk of bradycardia due to drug accumulation in elderly patients or those with unstable cardiovascular systems (Guo, 2023). Secondly, propofol can lower heart rate by inhibiting sympathetic nervous activity during anesthesia and sedation (Robinson et al., 1997). Conversely, ciprofol is able to maintain sympathetic nervous activity, thereby supporting cardiovascular compensatory responses during the perioperative period (Liao et al., 2025). Finally, propofol reduces myocardial contractility, cardiac output, and blood pressure by inhibiting L-type calcium channels, which can trigger vagal reflexes that lead to decreased heart rate (Fassl et al., 2011). However, ciprofol has a minimal effect on L-type calcium channels, suggesting that it has a weaker impact on myocardial contractility and cardiac output (Wang et al., 2023). As a result, compared with propofol, ciprofol exerts less influence on blood pressure and vagal reflexes during anesthesia, which may account for its lower incidence of bradycardia.

Interestingly, Liu et al. (2024) and Qi et al. (2024), reported no significant difference in the incidence of bradycardia between ciprofol and propofol during endoscopic sedation (risk difference (RD) 0.00, 95% CI -0.04 to 0.04) and perioperative sedation (RR 0.84, 95% CI 0.6–1.16). This discrepancy may be related to the mean age of the participants, as the average age of the participants in the studies by Liao et al. (2022) and Cheng et al. (2025) and our study was over 65 years, while the studies by Liu et al. (2024) and Qi et al. (2024) included participants across all age groups. This implies that the beneficial effect of ciprofol in reducing bradycardia may be restricted to the older population. Several reasons can explain this phenomenon: For one thing, aging causes a series of degenerative changes in the structure and function of the heart, such as a reduction in the number and fibrosis of pacemaker cells in the sinus node, atrioventricular node, hippocampal bundle, and left bundle branch (Obas and Vasan, 2018; Choi et al., 2021; Gazoti Debessa et al., 2001); For another, aging changes the balance of the autonomic nervous system, with increased parasympathetic tension and decreased sympathetic responsiveness (Jakovljevic, 2018).

In previous studies, Liu et al. (2024) reported that ciprofol reduced the incidence of hypotension during endoscopy compared to propofol (RR 0.73, 95% CI 0.58–0.92), while Cheng et al. (2025) found a similar reduction in hypotension risk with ciprofol in perioperative sedation (OR 0.48, 95% CI 0.32–0.72). Likewise, Chen et al. (2025) (RR 0.72, 95% CI 0.56–0.94) and Qi et al. (2024) (RR 0.64, 95% CI 0.52–0.77) also noted that ciprofol decreased the risk of hypotension compared to propofol, further supporting the benefits of ciprofol in this regard. Our meta-analysis also demonstrated a statistically significant difference in the benefits of ciprofol concerning hypotension; however, sensitivity analysis and TSA indicated that this result was unstable and lacked conclusiveness. Specifically, the sensitivity analysis suggested that the findings related to hypotension were not robust, as the results became insignificant after excluding the study by He et al. (Liao et al., 2022) (RR 0.79, 95% CI 0.59–1.07, P = 0.12). The observed high sensitivity to hypotension may be influenced by the small sample size. In this meta-analysis, the total number of patients with hypotension was only 778, which decreased to 628 after excluding He et al.'s study (Liao et al., 2022). This small sample size may not adequately reflect the significance of minor differences. Moreover, this speculation was corroborated by the TSA, which indicated a lack of conclusive benefits regarding hypotension with ciprofol use. Therefore, the effects of ciprofol versus propofol on the risk of hypotension in patients undergoing ERCP warrant further evaluation in future clinical trials.

### 4.3 Effects on the respiratory system

The meta-analysis demonstrated that compared to propofol, ciprofol significantly reduced the incidence of respiratory depression and hypoxemia without a significant effect on choking cough. Leave-one-out sensitivity analyses indicated that the results for respiratory depression, hypoxemia, and choking cough were robust. Subsequent TSA revealed conclusive differences in the incidence of respiratory depression and hypoxemia between the ciprofol and propofol groups. In previous meta-analyses, Qi et al. (2024) found a significant reduction in respiratory adverse events with ciprofol compared to propofol (RR 0.44, 95% CI 0.35–0.55), and Chen et al. (2025) reported benefits in terms of respiratory depression (RR 0.29, 95% CI 0.19–0.43) and hypoxemia (RR 0.38, 95% CI 0.26–0.55). Similarly, Cheng et al. (2025) highlighted the advantages of ciprofol in reducing the risk of respiratory depression (OR 0.21, 95% CI 0.15–0.30) and hypoxemia (OR 0.29, 95%CI 0.20–0.43). These results are concurrent our findings and suggest that the respiratory side effects of ciprofol are less severe than those of propofol. The difference in respiratory depression between the two drugs is related to the activation of GABA receptor subtypes. Specifically, ciprofol mainly binds the α1, β2, and γ2 subtypes of GABA receptors to exert sedative effects, whereas propofol binds all α, β, and γ subtypes (Jiang et al., 2021; Liao et al., 2022). However, the β3 subtype, a key target of propofol, has a direct inhibitory effect on the function of respiratory-related brain nuclei, which leads to a greater tendency of respiratory depression and hypoxemia with propofol (Jiang et al., 2021). Additionally, our meta-analysis found no significant difference in the incidence of choking cough between the ciprofol and propofol groups, which has not been reported in previous meta-analyses.

### 4.4 Effects on the nervous and digestive system

The meta-analysis revealed that compared to propofol, ciprofol significantly reduced injection pain, with no significant effect on involuntary movements. Subsequent TSA showed a conclusive benefit of ciprofol with regard to injection pain. In previous meta-analyses, Chen et al. (2025) (RR 0.13, 95% CI 0.09–0.20), Cheng et al. (2025) (OR 0.08, 95% CI 0.05–0.15), Qi et al. (Qi et al., 2024) (RR 0.12, 95% CI 0.08–0.17), and Liu et al. (Liu et al., 2024) (RD −0.34, 95% CI -0.48 to - 0.19) reported that the incidence of injection pain was significantly lower with ciprofol than with propofol, supporting our findings. Moreover, sensitivity analyses showed heterogeneity in injection pain, originating from a study by Wang et al. (2024), which used lower induction and maintenance doses of ciprofol. However, after excluding the study by Wang et al. (2024), the difference in the incidence of injection pain remained significant (RR 0.10, 95% CI 0.05–0.19, P < 0.00001, I^2^ = 0%), suggesting that the results were robust. The difference in the incidence of injection pain between the two drugs may be related to their water solubility. The methyl group of propofol is replaced with a cyclopropyl group in ciprofol, resulting in higher lipid solubility and lower water solubility (Guo, 2023). Consequently, ciprofol is less soluble in body fluids, which reduces irritation of the venous vascular lining to some extent (Sawant et al., 2021).

Interestingly, although Liu et al. (2024) concluded that there was no significant difference between ciprofol and propofol in the incidence of involuntary movements (RR 0.84, 95% CI 0.64–1.10), Chen et al. (2025) claimed that ciprofol reduced the incidence of involuntary movements (RR 0.73, 95% CI 0.56–0.96). This contradiction may be attributed to the difference inclusion criteria for participants, as the present study and that of Liu et al. (2024) only included participants who underwent endoscopic surgery, whereas Chen et al. (2025) included participants who underwent all types of surgery. The complexity and variety of surgery types could be a potential confounding variable, leading to conflicting results on involuntary movements. Considering that there was no heterogeneity in the results of involuntary movements in this meta-analysis and that the findings were robust, the risk of involuntary movements may be comparable between ciprofol and propofol.

Furthermore, this study found that the incidence of nausea and vomiting was comparable between ciprofol and propofol, and sensitivity analyses indicated that this result was robust. In previous meta-analyses, Chen et al. (2025) (RR 0.69, 95% CI 0.43–1.11) and Liu et al. (2024) (RR -0.02, 95% CI -0.06 to 0.02) both reported that ciprofol did not reduce the occurrence of nausea and vomiting compared to propofol, supporting our findings. Considering the non-heterogeneity and robustness of results, ciprofol may have no additional benefit in reducing the risk of nausea and vomiting.

### 4.5 Discovery and inspiration

The demonstrated advantages of ciprofol over propofol in reducing the incidence of respiratory and circulatory depression, and injection pain, suggest that this agent could significantly influence the current sedation protocols for ERCP, particularly in vulnerable patient populations. In clinical practice, older patients and those with pre-existing cardiovascular conditions often face heightened risks associated with traditional sedatives, such as propofol, which can exacerbate hypotension and respiratory depression. The findings of this meta-analysis indicate that ciprofol may offer a safer alternative, enabling clinicians to tailor anesthesia plans to minimize hemodynamic and respiratory complications. Ultimately, this study may pave the way for future clinical studies aimed at optimizing the application of ciprofol and fostering more personalized, risk-averse sedation practices for ERCP, and potentially other endoscopic procedures.

Notably, the RCTs included in this analysis employed varying exclusion criteria. For instance, some studies excluded morbidly obese patients, individuals with uncontrolled diabetes, or those with significant cardiopulmonary comorbidities, while others excluded patients undergoing emergency surgery, those with bradycardia, or hypoxemia. Consequently, the current evidence primarily reflects outcomes in relatively low-to moderate-risk populations and may not be directly generalizable to patients with severe comorbidities or unstable clinical conditions. Therefore, although our findings suggest that ciprofol could be a safer alternative to propofol for ERCP sedation in populations similar to those studied, caution should be exercised when applying these results to higher-risk groups. Further research is necessary to evaluate the safety and efficacy of ciprofol specifically in vulnerable populations, such as morbidly obese patients, individuals with poorly controlled chronic diseases, or patients requiring emergency procedures. In clinical practice, individual risk assessment remains crucial, and sedative choice should be carefully tailored to each patient’s comorbidities and procedural context.

### 4.6 Limitations and prospects

Although this study provides an evidence-based medical reference for the use of ciprofol in ERCP, it has some limitations. First, there may have been a potential selection bias, as four studies did not report allocation concealment. Future clinical trials should improve the methodological rigor to achieve low bias in clinical data reporting and analysis. Second, ciprofol is currently only approved for clinical sedation by the National Medical Products Administration of China. Therefore, all the included participants were Chinese. However, the East Asian population has a unique pharmacogenetic profile (Gu et al., 2023), and there are significant differences in the gene polymorphisms of uridine diphosphate glucuronosyltransferase 1A9 (UGT1A9), which metabolizes ciprofol, among different ethnic groups (Hou et al., 2024; Mehlotra et al., 2007). This limits the external validity of the study results, especially for Western or multi-ethnic populations with different comorbidities and pharmacogenetic profiles undergoing ERCP. In the future, global multicenter collaborative studies should be actively conducted to include patients from different regions and ethnicities to enhance the generalizability. Third, this study could not investigate the effects of ciprofol in patients with different comorbidities because no significant stratification by comorbidities was evident among the included studies. Therefore, future studies are needed to explore the effects of ciprofol in patients with different comorbidities, especially in those with combined respiratory or cardiovascular diseases. Fourth, not all studies reported the outcomes of interest. For example, only three to five studies reported on circulatory and respiratory outcomes, such as bradycardia, hypotension, respiratory depression, hypoxemia, and choking cough. This may reduce the precision and credibility of the results. Fifth, this study only explored the benefits of ciprofol relative to propofol, but did not determine the optimal dose of ciprofol. Therefore, future rigorous RCTs are needed to explore the optimal dose of ciprofol in ERCP and provide guidance for clinical anesthesia. Sixth, although we reviewed and discussed the pharmacodynamic and pharmacokinetic reasons for the differences in safety between ciprofol and propofol as much as possible, the limited literature and evidence restricted the in-depth analysis of the underlying mechanisms. Future studies should provide molecular modeling, receptor binding analysis, or mechanistic references to support key pharmacological claims.

## 5 Conclusion

Ciprofol has been shown to reduce the incidence of bradycardia, respiratory depression, hypoxemia, and injection pain compared to propofol; however, its impact on the incidence of hypotension requires further evaluation. This suggests that ciprofol may be a safer alternative to propofol for use in ERCP procedures. Nevertheless, future studies are needed to determine the safety, efficacy, and optimal dosing of ciprofol in diverse patient populations, including those with complex comorbidities. Such investigations would support its wider application in ERCP as well as other surgical procedures, including gastrointestinal and ophthalmic surgeries.

## Data Availability

The original contributions presented in the study are included in the article/Supplementary Material, further inquiries can be directed to the corresponding authors.

## Abbreviations

BMI, body mass index; CI, confidence interval; CNKI, China National Knowledge Infrastructure; CSTJ, China Science and Technology Journal Database; ERCP, endoscopic retrograde cholangio-pancreatography; RCTs, randomized controlled trials; RR, risk ratio; TSA, trial sequential analysis; GABA, gamma-aminobutyric acid; ASA, American Society of Anesthesiologists; PROSPERO, Prospective Register of Systematic Reviews; PRISMA, Preferred Reporting Items for Systematic Reviews and Meta-Analyses; GRADE, Grading of Recommendations, Assessment, Development, and Evaluation.

## References

[B1] Azimaraghi O. Bilal M. Amornyotin S. Arain M. Behrends M. Berzin T. M. (2023). Consensus guidelines for the perioperative management of patients undergoing endoscopic retrograde cholangiopancreatography. Br. J. Anaesth. 130, 763–772. 10.1016/j.bja.2023.03.012 37062671

[B2] Chen W. Xu Y. Zeng Y. Xing G. (2025). A meta-analysis and systematic review based on perioperative management of elderly patients: is ciprofol an alternative to propofol? Eur. J. Clin. Pharmacol. 81, 111–121. 10.1007/s00228-024-03782-7 39565391

[B3] Cheng X. Zhang P. Jiang D. Fang B. Chen F. (2025). Safety and efficacy of ciprofol versus propofol for gastrointestinal endoscopy: a meta-analysis. BMC Gastroenterol. 25, 130. 10.1186/s12876-025-03734-0 40033212 PMC11877735

[B4] Cheriyan D. G. Byrne M. F. (2014). Propofol use in endoscopic retrograde cholangiopancreatography and endoscopic ultrasound. World J. Gastroenterol. 20, 5171–5176. 10.3748/wjg.v20.i18.5171 24833847 PMC4017032

[B5] Choi S. Baudot M. Vivas O. Moreno C. M. (2021). Slowing down as we age: aging of the cardiac pacemaker’s neural control. GeroScience 44, 1–17. 10.1007/s11357-021-00420-3 34292477 PMC8811107

[B6] Ding G. Wang L. Zhao W. Diao Y. Song D. (2024). Comparison of the efficacy and safety of ciprofol and propofol for ERCP anesthesia in older patients: a single-center randomized controlled clinical study. J. Clin. Anesth. 99, 111609. 10.1016/j.jclinane.2024.111609 39288685

[B7] Fassl J. High K. M. Stephenson E. R. Yarotskyy V. Elmslie K. S. (2011). The intravenous anesthetic propofol inhibits human L-type calcium channels by enhancing voltage-dependent inactivation. J. Clin. Pharmacol. 51, 719–730. 10.1177/0091270010373098 20547772

[B8] Gao R. Liu L. (2024). Evaluation of anesthetic effect and safety of ciprofol in elderly patients undergoing endoscopic retrograde cholangiopancreatography choledochotomy. J. Chin. Foreign Med. Pharm. Res. 3, 9–12. 10.3969/j.issn.2096-6229.2024.21.003

[B9] Gazoti Debessa C. R. Mesiano Maifrino L. B. Rodrigues de Souza R. (2001). Age related changes of the collagen network of the human heart. Mech. Ageing Dev. 122, 1049–1058. 10.1016/S0047-6374(01)00238-X 11389923

[B10] Gu J. Q. Jiang L. Xu J. Y. Wang H. Wei Y. L. Li C. X. (2023). Genetic structure of East asians based on high-density SNP data. Prog. Biochem. Biophys. 50, 2739–2752. 10.16476/j.pibb.2022.0441

[B11] Guo M. (2023). Analysis of the mechanism of action of cyclopol in clinical application. ACM 13, 14569–14573. 10.12677/ACM.2023.1392037

[B12] He K. Liu Y. P. Wu J. Yang X. H. (2024). Application of ciprofol in painless endoscopic retrograde cholangiopancreatography. J. Tongji Univ. 45, 210–215. 10.12289/j.issn.2097-4345.23194

[B13] Hou L. Zhao Y. Zhao S. Zhang X. Yao X. Yang J. (2024). Ciprofol is primarily glucuronidated by UGT1A9 and predicted not to cause drug-drug interactions with typical substrates of CYP1A2, CYP2B6, and CYP2C19. Chem. Biol. Interact. 387, 110811. 10.1016/j.cbi.2023.110811 37993078

[B14] Huang J. Zhang J. S. Jiang P. W. Ma F. D. Yang M. Y. Yang Y. (2023). Progress on clinical application of ciprofol. Chin. J. Anesth. 43, 1149–1152. 10.3760/cma.j.cn131073.20230323.00926

[B15] Jakovljevic D. G. (2018). Physical activity and cardiovascular aging: physiological and molecular insights. Exp. Gerontol. 109, 67–74. 10.1016/j.exger.2017.05.016 28546086

[B16] Jiang J. Jiao Y. Gao P. o. Yin W. Zhou W. Zhang Y. (2021). Propofol differentially induces unconsciousness and respiratory depression through distinct interactions between GABA_A_ receptor and GABAergic neuron in corresponding nuclei. ABBS 53, 1076–1087. 10.1093/abbs/gmab084 34137445

[B17] Khamaysi I. Taha R. (2020). ERCP for severe acute cholangitis: the earlier, the better. Turk J. Gastroenterol. 31, 78–79. 10.5152/tjg.2020.19103 32009619 PMC7075686

[B18] Liao J. Li M. Huang C. Yu Y. Chen Y. Gan J. (2022). Pharmacodynamics and pharmacokinetics of HSK3486, a novel 2,6-disubstituted phenol derivative as a general anesthetic. Front. Pharmacol. 13, 830791. 10.3389/fphar.2022.830791 35185584 PMC8851058

[B19] Liao M. Wu X. R. Hu J. N. Lin X. Z. Zhao T. Y. Sun H. (2025). Comparative effective dose of ciprofol and propofol in suppressing cardiovascular responses to tracheal intubation. Sci. Rep. 15, 1822. 10.1038/s41598-025-85968-2 39805976 PMC11730606

[B20] Liu J. Hong A. Zeng J. Liang X. (2024). The efficacy of ciprofol versus propofol on anesthesia in patients undergoing endoscopy: a systematic review and meta-analysis of randomized controlled trials. BMC Anesthesiol. 24, 359. 10.1186/s12871-024-02721-4 39379828 PMC11460030

[B21] Lu M. Liu J. Wu X. Zhang Z. (2023). Ciprofol: a novel alternative to propofol in clinical intravenous anesthesia? Biomed. Res. Int. 2023, 7443226. 10.1155/2023/7443226 36714027 PMC9879693

[B22] Mehlotra R. K. Bockarie M. J. Zimmerman P. A. (2007). Prevalence of UGT1A9 and UGT2B7 nonsynonymous single nucleotide polymorphisms in west african, papua new guinean, and north American populations. Eur. J. Clin. Pharmacol. 63, 1–8. 10.1007/s00228-006-0206-z 17115150 PMC2577308

[B23] Mo C. F. (2024). Effectiveness and safety of ciprofolcombined with sufentanil and dexmedetomidine in elderly people undergoing endoscopic retrograde cholangiopancreatography. *Shan Tou University* .

[B24] Obas V. Vasan R. S. (2018). The aging heart. Clin. Sci. (Lond) 132, 1367–1382. 10.1042/CS20171156 29986877

[B25] Page M. J. McKenzie J. E. Bossuyt P. M. Boutron I. Hoffmann T. C. Mulrow C. D. (2021). The PRISMA 2020 statement: an updated guideline for reporting systematic reviews. BMJ 372, n71. 10.1136/bmj.n71 33782057 PMC8005924

[B26] Pal P. Ramchandani M. (2024). Management of ERCP complications. Best. Pract. Res. Clin. Gastroenterol. 69, 101897. 10.1016/j.bpg.2024.101897 38749576

[B27] Phillips A. T. Deiner S. Mo Lin H. Andreopoulos E. Silverstein J. Levin M. A. (2015). Propofol use in the elderly population: prevalence of overdose and association with 30-day mortality. Clin. Ther. 37, 2676–2685. 10.1016/j.clinthera.2015.10.005 26548320 PMC5864105

[B28] Qi J. Zhang L. Meng F. Yang X. Chen B. Gao L. (2024). Comparative effects of ciprofol and propofol on perioperative outcomes: a systematic review and meta-analysis of randomized controlled trials. Braz J. Anesthesiol. 75, 844578. 10.1016/j.bjane.2024.844578 39608601 PMC11699592

[B29] Qin L. Ren L. Wan S. Liu G. Luo X. Liu Z. (2017). Design, synthesis, and evaluation of novel 2,6-disubstituted phenol derivatives as general anesthetics. J. Med. Chem. 60, 3606–3617. 10.1021/acs.jmedchem.7b00254 28430430

[B30] Rastogi A. Campbell D. R. (2006). ERCP in the elderly: how safe is it? (marathons, marathon ERCPs, and marathon ERCPs in the elderly). Gastrointest. Endosc. 63, 956–958. 10.1016/j.gie.2005.11.022 16733109

[B31] Robinson B. J. Ebert T. J. O’Brien T. J. Colinco M. D. Muzi M. (1997). Mechanisms whereby propofol mediates peripheral vasodilation in humans. Sympathoinhibition or direct vascular relaxation? Anesthesiology 86, 64–72. 10.1097/00000542-199701000-00010 9009941

[B32] Sawant A. Kamath S. Kg H. Kulyadi G. P. (2021). Solid-in-oil-in-water emulsion: an innovative paradigm to improve drug stability and biological activity. AAPS PharmSciTech 22, 199. 10.1208/s12249-021-02074-y 34212274 PMC8249250

[B33] Schmalz M. J. Geenen J. E. (1999). Therapeutic pancreatic endoscopy. Endoscopy 31, 88–94. 10.1055/s-1999-14119 10082415

[B34] Shen Y. J. Diao Y. G. Sun Y. J. (2024). Study on the safety of ciprofol combined with alfentanil for ERCP anesthesia in elderly patients. Pract. Pharm. Clin. Remedies 27, 22–25. 10.14053/j.cnki.ppcr.202401005

[B35] Shimizu H. Homma Y. Norii T. Japanese Procedural Sedation and Analgesia Registry investigators (2021). Incidence of adverse events among elderly vs non-elderly patients during procedural sedation and analgesia with propofol. Am. J. Emerg. Med. 44, 411–414. 10.1016/j.ajem.2020.04.094 32409101

[B36] Short C. E. Bufalari A. (1999). Propofol anesthesia. Vet. Clin. North Am. Small Anim. Pract. 29, 747–778. 10.1016/s0195-5616(99)50059-4 10332821

[B37] Talukdar R. (2016). Complications of ERCP. Best. Pract. Res. Clin. Gastroenterol. 30, 793–805. 10.1016/j.bpg.2016.10.007 27931637

[B38] Task Force on Guidelines on Clinical Application of Ciprofol. (2023). Guidelines on clinical application of ciprofol. Chin. J. Anesthesiol. 43, 769–772. 10.3760/cma.j.cn131073.20230711.00701

[B39] Wang C. Dong X. Zhao K. (2023). Effect of ciprofol combined with afentanil in colonoscopy of senile patients. J. Clin. Anesthesiol., 550–552.

[B40] Wang J. Wang R. Ma X. Zhu W. Zhang B. Ma Y. (2024). Comparative efficacy of ciprofol and propofol in reducing respiratory depression during ERCP anesthesia: a randomized controlled trial. BMC Anesthesiol. 24, 404. 10.1186/s12871-024-02791-4 39516741 PMC11546404

[B41] Zhao W. T. Cui B. Xu Z. Z. Song D. D. (2023). Efficacy of ciprofol in elderly patients undergoing endoscopic retrograde cholangiopancreatography. J. Clin. Anesthesiol. 39, 610–613. 10.12089/jca.2023.06.010

